# Governor Vessel Moxibustion Therapy Improves Microbiota Structure in Ankylosing Spondylitis Patients

**DOI:** 10.1155/2021/9370758

**Published:** 2021-12-20

**Authors:** Guodong Sun, Qingbo Wang, Shengnan Cao, Haidong Xu, Yan Zhao

**Affiliations:** ^1^Academy of Medical Engineering and Translational Medicine, Tianjin University, Tianjin 300072, China; ^2^Department of Rehabilitation Medicine, Affiliated Hospital of Shandong Academy of Medical Sciences, Jinan, Shandong 250031, China; ^3^Shandong First Medical University, Jinan, Shandong 250117, China; ^4^Shandong University of Traditional Chinese Medicine, Jinan, Shandong 250355, China; ^5^Biomedical Sciences College & Shandong Medicinal Biotechnology Centre, Shandong First Medical University & Shandong Academy of Medical Sciences, Jinan, Shandong 250062, China

## Abstract

**Background:**

Clinical studies have shown that ankylosing spondylitis (AS) could be significantly improved by Governor Vessel moxibustion (GVM) therapy.

**Objective:**

Study whether GVM therapy alleviates the clinical symptoms of AS by modulating intestinal microbiota.

**Methods:**

A total of 9 AS patients and 9 paired healthy individuals were enrolled, and GVM therapy was provided to the AS patients. Stool, urine, and saliva samples from the healthy individuals and the AS patients before and after GVM therapy were collected, and 16S rRNA gene sequencing was performed for microbiota analysis.

**Results:**

We found that GVM therapy can significantly alleviate the symptoms of AS, such as diarrhea, abdominal pain, and bloating. GVM therapy also decreased the abundances of *Bacteroides* and *Prevotella* while increasing the abundances of beneficial bacteria, such as *Lactobacillus*, in the gut microbiota of the AS patients. The analyses for AS clinical data and microbial abundances in AS patients revealed their multiple significant correlations (*P* < 0.01); for example, an unclassified crystal was positively correlated with *AF12* and *Delftia*, monocyte had a negative correlation with *Scardovia*, and human leukocyte antigen-B27 was negatively correlated with *Catenibacterium*, *Coprococcus*, and *Oscillospira*.

**Conclusions:**

Overall, these findings demonstrate that GVM therapy can alleviate AS clinical symptoms, and at the same time, it improves the microbial structure of microbiota in AS patients. This trial is registered with Chinese Clinical Trial Registry ChiCTR2100051907.

## 1. Introduction

Ankylosing spondylitis (AS) is a chronic inflammatory systemic autoimmune disease characterized by the inflammation of the axial skeleton, peripheral joints, and the attachments of ligaments and entheses. AS can further progress into excessive bone ankylosis and syndesmophyte formation that slowly bridges and ultimately fuses the joints, causing stiffness and pain [1]. The prevalence of AS ranges from 0.2% to 0.4% in the Chinese population, and it is approximately 0.5% in the USA [2]. AS mainly impairs the physical activities, working ability, and life quality of the patients, which consequently imposes a considerable burden on both the patients and the entire society [3]. Moreover, evidences have shown that nearly 10% of AS patients could develop subclinical gut inflammation with diarrhea-related complications [4].

At present, a wide range of treatments is available for AS therapeutics, including pharmacological therapy, manual and physical therapies, and even interventional approaches, such as radiofrequency, pharmacology, and surgery. However, the therapeutic outcomes of these approaches are not satisfactory in many cases, and they often introduce side effects as well [5]. Therefore, a convenient, effective, and low-cost treatment is urgent to be developed.

Moxibustion is considered one of the effective traditional Chinese therapies for AS management, and it has been investigated based on several animal models of inflammatory bowel disease (IBD) [6]. Moxibustion could improve microscopic colitis in rats by decreasing the level of tumor necrosis factor [7]. It was also found that moxibustion could reduce the relative DNA abundances of *Prevotella*, *Bacteroides*, and *Clostridium XI*, while increasing those of *Lactobacillus* and *Clostridium* XIVa in the gut intestine, and meanwhile, it improved the *α*-diversity of gut microbiota in a rat model of irritable bowel syndrome (IBS) [8]. Hence, therapy by moxibustion is one of the promising approaches for AS treatment.

The aim of this study was to determine the effectiveness of moxibustion on gut microbiota and the potential roles of intestinal microbes in the pathogenesis of AS. Therefore, the clinical outcomes of Governor Vessel moxibustion (GVM) treatment in AS patients were assessed. Simultaneously, 16S rRNA amplicon sequencing was performed to evaluate the modulatory functions of GVM on gut microbiota in AS patients and healthy individuals.

## 2. Materials and Methods

### 2.1. Patient Enrollment and Sample Collection

The study was approved by the Ethics Committee of the Affiliated Hospital of Shandong Academy of Medical Sciences (2018-02). All individuals provided their informed consent for inclusion before they participated in the study. A total of 18 patients were enrolled including 9 patients with AS, who fulfilled the AS New York diagnostic criteria as revised by the American College of Rheumatology [9], and 9 healthy individuals serving as the controls, who had never been diagnosed with AS, IBD, or any other autoimmune diseases. Clinical information of the participants (gender, age, and duration) was collected, and BASFI, BASDAI, morning stiffness, occipital-wall distance, Schober test, finger-to-ground distance, thorax active level, and VAS were calculated.

Saliva samples were collected in 50 mL sterile tubes from each patient, between 7 and 9 am. All the patients were asked to refrain from drinking and eating and hygiene-related procedures for at least 2 hours prior to the sample collection. Fecal samples were collected in stool collection tubes prefilled with absolute ethanol from each patient. Urine samples were collected in 50 mL sterile tubes from each patient in the morning. The samples were immediately frozen and stored at -80°C for further detection and analysis. Data of clinical diagnosis and blood examination were obtained from the hospital.

### 2.2. GVM Treatment

All the AS patients (AS group) and healthy individuals (HC group) were subjected to GVM treatment through sandwiched moxibustion (Supplemental Figure 1). Briefly, the main ingredients, white peony and musk, combined with the compositions of traditional Chinese medicines, such as Chuanxiong and Haifengteng, were milled into powder. The patient was lying on a bed in prone position. Approximately 30 g of the medicine powder was mixed with ginger juice and evenly sprinkled on the spinal section of the Governor Vessel of the patient. The treatment area was covered by a mulberry paper (10 cm × 50 cm), with ginger paste placed in the shape of the trapezium (7 cm × 4 cm) from Dazhui (DU 14) to Yaoshu (DU 2). Then, moxa was kneaded into ellipses (5 cm × 3 cm), placed along the ginger paste, and allowed to burn until self-extinguished. The moxa cone was replaced twice. Eventually, the treatment area was gently wiped with a towel or gauze. The duration of each treatment was approximately 1.5-2 h. The patients were treated once a week and four times a course.

### 2.3. DNA Extraction and 16S rRNA Sequencing

Each individual sample was centrifuged at 12,000 × g for 15 minutes at 4°C for DNA extraction by using the Qiagen DNeasy Blood & Tissue Kit (Qiagen, Valencia, CA, USA) according to the manufacturer's instruction, and the extracted DNA was quantified by using NanoDrop 2100 (Thermo Scientific).

The paired-end 16S sequence dataset was joined into single-sequence reads and filtered by the FLASH method described by Magoč and Salzberg [10]. All the sequence analyses were performed by using Quantitative Insights into Microbial Ecology (QIIME) software suite version 1.9.1 [11], according to the tutorial (http://qiime.org/) with slight modifications. Chimeric sequences were removed by usearch61 with de novo models [12]. Then, the sequences were clustered against the Greengenes 13_5 ribosomal database (http://greengenes.secondgenome.com/downloads) at 97% identity. The sequences not matching with any entries in the reference were subsequently clustered into de novo OTUs at 97% similarity with UCLUST. Taxonomy was assigned to all the OTUs by using the Ribosomal Database Project classifier [13] in QIIME and the Greengenes reference dataset. Rarefaction and rank abundance curves were calculated from OTU tables by using *α*-diversity and rank abundance scripts in the QIIME pipeline. The hierarchical clustering based on population profiles of the most common and abundant taxa was performed by using the unweighted pair group method with arithmetic mean clustering based on the distance matrix of OTU abundances. The Newick formatted tree was obtained by using the QIIME package.

### 2.4. Statistical Analysis

PCoA was performed for the evenly sampled OTU abundances based on unweighted UniFrac distances. The Shannon index representing *α*-diversity was calculated. All the statistical analyses were performed by using SPSS 22.0. The clinical indicators were expressed as ($\bar{X}\pm \text{S}$), which were consistent with the data of normal distribution and homogeneity of variance. The comparison between groups was conducted by a *t*-test, and *P* < 0.05 was considered statistically significant.

## 3. Results

### 3.1. Clinical Indicators

The 9 AS patients included 8 males and 1 female, with their age ranged from 34 to 49 years old (41.89 ± 7.29) and the disease duration ranged from 4 to 20 years (12.67 ± 6.46). The healthy group included 3 males and 6 females, with their age ranged from 35 to 68 years old (47.56 ± 10.18). The AS patients before GVM treatment were defined as “before,” and those after GVM treatment were defined as “after.” The parameters of disease activities, including bath AS disease activity index (BASDAI), bath AS function index (BASFI), Schober test, visual pain analog scale (VAS), morning stiffness, finger-to-ground distance, thorax active level, and occipital-wall distance, are summarized in Table 1. The differences between “before” and “after” groups were statistically significant (*P* < 0.05).

### 3.2. Microbiota Diversity in Saliva, Stool, and Urine

**Figure 1:**
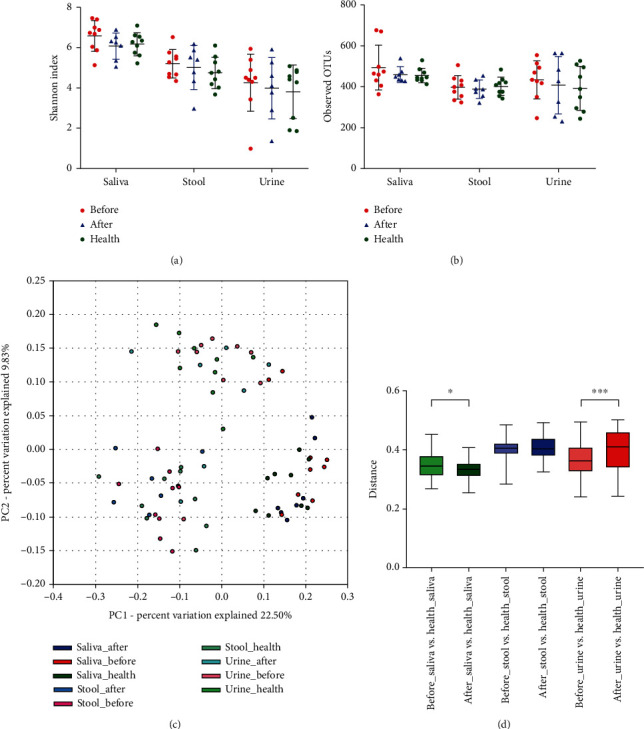
Effects of Governor Vessel moxibustion (GVM) treatment on the composition of human saliva, stool, and urine microbiota. (a) The Shannon index for *α*-diversity; (b) rarefaction analysis of observed OTUs; (c) principal coordinate analysis for *β*-diversity; (d) unweighted UniFrac distances. Before = ankylosing spondylitis patients before GVM treatment; after = ankylosing spondylitis patients after GVM treatment; health = healthy individuals. ^∗^*P* < 0.05; ^∗∗∗^*P* < 0.001.

We compared the Shannon index (Figure 1(a)) and the observed operational taxonomic units (OTUs) (Figure 1(b)) in saliva, stool, and urine samples across groups. In comparison with the AS patients before GVM, the *α*-diversities of the three types of microbiotas were all decreased by the GVM treatment, while the differences were not significant (FDR > 0.05). Moreover, the *α*-diversity of stool microbiota in the AS patients was recovered back to be similar with that in the healthy individuals (FDR > 0.05).

We further evaluated the *β*-diversity among the three groups by principal coordinate analysis (PCoA), based on the unweighted UniFrac distance (Figure 1(c)). The PCoA analysis revealed unique clusters of microbial communities in different groups (*P* = 0.001, *R*^2^ = 0.2573). For unweighted PCoA analysis, PC1 explained 22.50% and PC2 explained 9.83% of the total variation. For saliva microbiota, the healthy individuals had a closer distance with the patients after GVM treatment than that with the patients before GVM (*P* = 0.0302). For urine microbiota, the healthy individuals had a farther distance with the patients after GVM treatment than that with the patients before GVM (*P* = 0.0022), whereas no significant change was observed in stool microbiota after GVM treatment (Figure 1(d)).

### 3.3. Microbial Community Composition of Saliva, Stool, and Urine Microbiota

**Figure 2:**
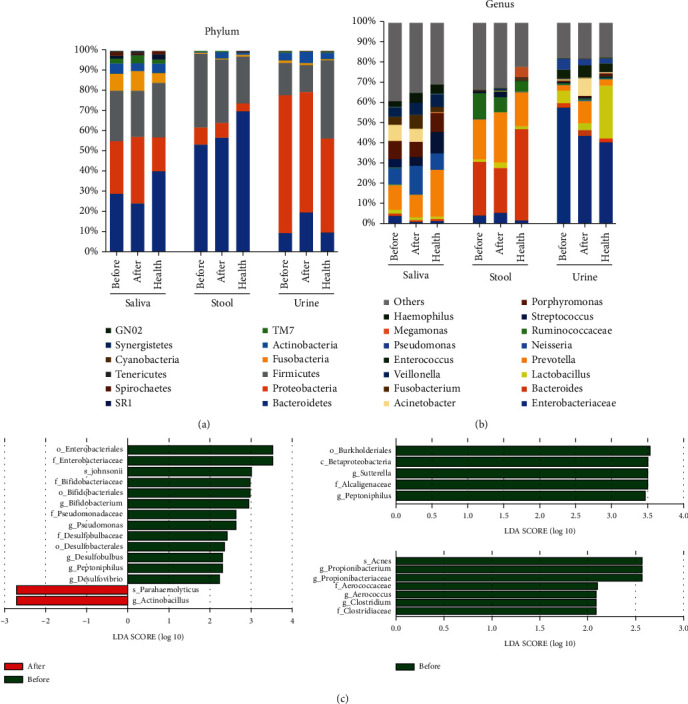
Taxonomic profiles and biomarkers of saliva, stool, and urine microbiota in healthy individuals and ankylosing spondylitis (AS) patients before and after Governor Vessel moxibustion (GVM) treatment. (a) Bar plots of taxonomic profiles at the phylum level. (b) Bar plots of taxonomic profiles at the genus level. (c) Histogram of the LDA scores, indicating the effective size and rank of each differentially abundant taxon (LDA > 2). Before = ankylosing spondylitis patients before GVM treatment; after = ankylosing spondylitis patients after GVM treatment; health = healthy individuals.

The overall microbial components of microbiota in the AS patients and healthy individuals were similar, with moderate differences found in the abundances of certain types of bacteria at the phylum and genus levels. Specifically, Bacteroidetes, Proteobacteria, Firmicutes, Fusobacteria, and Actinobacteria were the dominant phyla in the microbiota of the individuals in all groups (Figure 2(a)). The abundances of Bacteroidetes were 29.7% (before), 24.6% (after), and 40.0% (health) in the saliva microbiota; 52.5% (before), 54.6% (after), and 70.0% (health) in the stool microbiota; and 10.0% (before), 20.1% (after), and 10.4% (health) in the urine microbiota. The abundances of Proteobacteria were 24.1% (before), 30.6% (after), and 15.0% (health) in the saliva microbiota; 8.5% (before), 7.4% (after), and 2.0% (health) in the stool microbiota; and 66.0% (before), 58.9% (after), and 43.2% (health) in the urine microbiota. Meanwhile, the abundances of Bacteroidetes were 26.2% (before), 25.0% (after), and 28.0% (health) in the saliva microbiota; 37.0% (before), 33.0% (after), and 25.0% (health) in the stool microbiota; and 16.0% (before), 12.0% (after), and 37.0% (health) in the urine microbiota.

At the genus level, in comparison with the “before” group, AS patients after GVM treatment possessed higher abundances of *Prevotella* and *Porphyromonas* while lower abundances of *Neisseria* and *Streptococcus* in their saliva samples; higher abundance of *Bacteroides* and lower abundance of *Streptococcus* in their stool samples; and higher abundances of *Enterobacteriaceae*, *Lactobacillus*, and *Pseudomonas* while lower abundances of *Acinetobacter* and *Prevotella* in their urine samples (Figure 2(b)).

The LEfSe (linear discriminant analysis effect size) method was further used to evaluate the differences among the microbiota composition of the AS patients before and after GVM treatment and to identify significant biomarkers (LDA > 2). Multiple taxa were differentially abundant in the saliva samples from the AS patients before and after GVM treatment, including *Enterobacteriales*, *Enterobacteriaceae*, *Johnsonii*, *Bifidobacteriaceae*, *Bifidobacterium*, *Pseudomonadaceae*, *Pseudomonas*, *Desulfobulbaceae*, *Peptoniphilus*, *Desulfovibrio*, *parahaemolyticus*, and *Actinobacillus.* Meanwhile, *Burkholderiales*, *Betaproteobacteria*, *Sutterella*, *Alcaligenaceae*, and *Peptoniphilus* in the stool microbiota and acnes and *Propionibacterium*, *Propionibacteriaceae*, *Aerococcaceae*, *Aerococcus*, *Clostridium*, and *Clostridiaceae* in the urine microbiota were enriched in the AS patients before GVM treatment (Figure 2(c)).

### 3.4. Unique and Shared OTUs in Stool, Urine, and Saliva Microbiota

**Figure 3:**
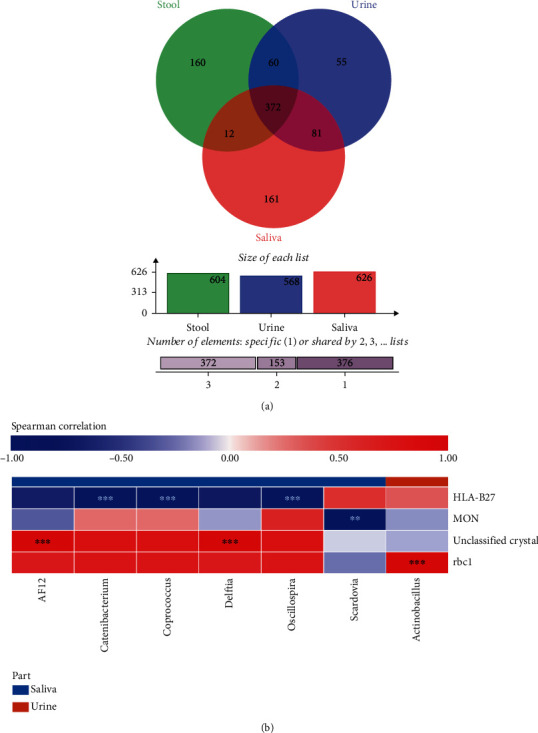
Relations among microbiota composition and the correlation between their clinical indicators and functions in ankylosing spondylitis patients. (a) Venn diagram depicting the unique or shared OTUs in stool, urine, and saliva microbiota. (b) Correlation between the expression of clinical indicators and microbiota functions in AS patients. rbc1 = red blood cell-1; HLA-B27 = human leukocyte antigen-B27; MON = monocytes. ^∗∗^*P* < 0.01;  ^∗∗∗^*P* < 0.001.

The OTUs shared by at least half of the saliva, stool, or urine samples from the AS patients are shown in Venn diagrams (Figure 3(a)). A total of 372 OTUs were shared among all the stools, urine, and saliva samples, while 160, 55, and 161 unique OTUs were detected in the stool, urine, and saliva samples, respectively. Thus, these results indicated the consistency of urine, saliva, and stool microbiota in the AS patients.

### 3.5. Indicator Bacteria Are Correlated with Microbiota Functions in AS Patients

Based on Spearman's rank correlation coefficient, we observed that unclassified crystal was positively correlated with AF12 or Delftia (*P* < 0.001). Simultaneously, a positive correlation was detected between red blood cells in urine and Actinobacillus (*P* < 0.001). In contrast, monocyte in blood was negatively correlated with Scardovia (*P* < 0.01), and human leukocyte antigen-B27 was negatively correlated with Catenibacterium, Coprococcus, or Oscillospira (*P* < 0.001) (Figure 3(b)).

## 4. Discussion

Recently, it has been demonstrated that the influence of gut microbiota extends beyond the gastrointestinal tract and reaches the systemic immune system. During the last decade, several studies have revealed the strong association between gut microbiome dysbiosis and AS [14, 15]. In this study, we have demonstrated a notable difference in the gut microbiome profiles between AS patients and healthy individuals. As a matter of fact, the spine is closely related to the Governor Vessel, and the disease progression of ankylosing spondylitis is induced by the deficiency and dysfunction of “Yang Qi,” the traveling of which in the human body depends on the Governor Vessel channel [16]. GVM therapy is based on the theory of acupoints on the Governor Vessel channel in traditional Chinese medicine, which is an effective and safe treatment for AS and is widely used in clinical practices [17]. Moreover, GVM therapy can also regulate gastrointestinal functions and relieve abdominal pain and diarrhea, though the underlying mechanism remains unknown. For the first time, we reveal the characteristics of gut microbiota in AS patients who accepted GVM treatment and analyze the connections among GVM, AS, and gut microbiota.

In this study, we employed 16S rRNA amplicon sequencing to determine the modulatory effects of moxibustion treatment on gut microbiome. The *α*-diversity of the gut microbiota in the AS patients and the healthy individuals was similar. Three of the highest dominant phyla in the microbiota of AS patients were Bacteroidetes, Proteobacteria, and Firmicutes. In normal condition, *Bacteroides* is not pathogenic, while it can become pathogenic under certain pathological conditions [18]. *Bacteroides* has a proinflammatory effect that promotes intestinal and systemic inflammation. *Lactobacillus* is frequently used as a probiotic, based on its capabilities in stimulating the production of a large quantity of anti-inflammatory interleukins to enhance the functions of the intestinal barrier, thus preventing the development of colitis in the gut intestine [19]. Several studies have shown that *Lactobacillus* GG could effectively mitigate IBS in both humans and rats [20]. Accordingly, we also revealed that moxibustion could reduce the abundance of *Bacteroides* and decrease *Prevotella* in saliva and increase *Lactobacillus* in stool.

GVM has comprehensive functions due to the combination of the integral effects of moxibustion and the specificity of Chinese herbal medicines (musk and white peony). Previous studies have demonstrated that the water-soluble components of musk are anti-inflammatory in various animal models, and the ingredients of musk glycoprotein have significant inhibitory effects on the chemotaxis of neutrophils [21, 22]. Meanwhile, glucosides extracted from peony have immunosuppressive effects on arthritis based on the rat model, which indicates the potential benefit of peony in the treatment of rheumatoid arthritis [23]. Accordingly, moxibustion combined with Chinese herbal medicines may also play an important role in the treatment of inflammation-related diseases. In addition, saliva, urine, and stool samples were collected from the same patient at the same time for analyzing the oral, urethral, and intestinal microbial community. The OTU data illustrate that *Bacteroides*, *Lactobacillus*, and *Prevotella* were present in all the samples, which demonstrates that the urine, saliva, and stool microbiota in these AS patients are consistent.

## 5. Conclusions

The related clinical score indicators of AS in the patients are improved by GVM. Dependent on the consistent microbial sequencing, we reveal that the microbiota in urine, stool, and saliva samples from AS patients are different from those in healthy individuals, but GVM can improve them by reducing the number of pathogenic bacteria while promoting beneficial bacteria. Overall, the GVM treatment contributes to suppressing the development/progression of AS through simultaneously alleviating its clinical symptoms and modulating the urethral, intestinal, and oral microbiome in the patients.

## Figures and Tables

**Table 1:** The clinical indicators for basic demographic characteristics.

Characteristic	Before	After	Health
Age (years)	41.89 ± 7.29	41.89 ± 7.29	47.56 ± 10.18
Male/female	8/1	8/1	3/6
Duration (years)	12.67 ± 6.46	12.67 ± 6.46	NA
Disease activity parameter, mean (SD)			
BASDAI (cm)	6.40 (0.25)	4.02 (0.64)^∗^	NA
BASFI (cm)	6.21 (0.27)	4.03 (0.30)^∗^	NA
Schober test (cm)	2.12 (0.27)	3.36 (0.33)^∗^	NA
VAS	6.03 (0.26)	4.00 (0.32)^∗^	NA
Morning stiffness (min)	41.00 (9.71)	17.33 (4.80)^∗^	NA
Finger-to-ground distance (cm)	30.67 (1.87)	19.33 (2.00)^∗^	NA
Thorax active level (cm)	1.49 (0.16)	3.07 (0.17)^∗^	NA
Occipital-wall distance (cm)	4.48 (0.53)	2.58 (0.45)^∗^	NA

Before = patients before GVM treatment; after = patients after GVM treatment; health = healthy individuals; NA = not applicable; SD = standard deviation; BASDAI = bath ankylosing spondylitis disease activity index; BASFI = bath ankylosing spondylitis function index; VAS = visual pain analog scale; ^∗^*P* < 0.05, “after” compared with “before”.

## Data Availability

The underlying data supporting the results of my study can be found by the correspondence author.

## Supplementary Materials

Supplementary Materials Supplemental Figure 1: schematic diagram of Governor Vessel moxibustion treatment.
