# Supplementary figures and images for: Governor Vessel Moxibustion Therapy Improves Microbiota Structure in Ankylosing Spondylitis Patients
Source: Dis Markers. 2021 Dec 20;2021:9370758. doi: 10.1155/2021/9370758 (PMC8712134; doi:10.1155/2021/9370758)

Ginger

Moxa

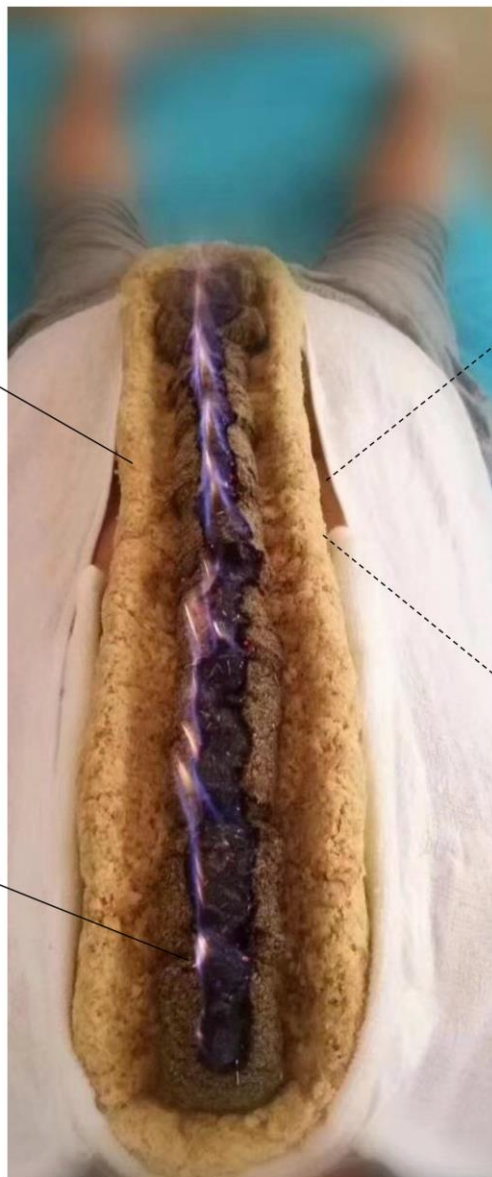

Traditional Chinese medicines

With white peony

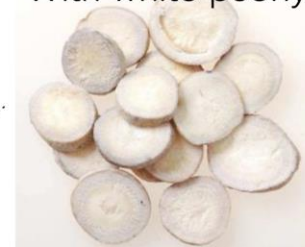

musk

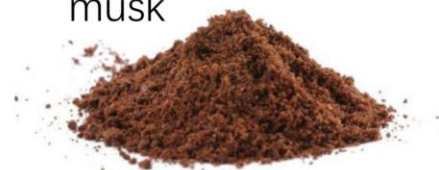

Chuanxiong

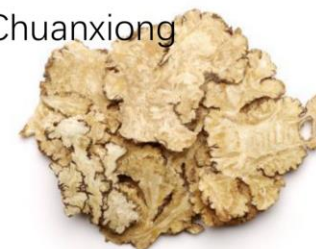

Haifengteng

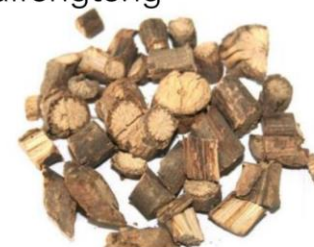

Supplement: Supplementary Materials — Supplemental Figure 1: schematic diagram of Governor Vessel moxibustion treatment. [file 9370758.f1.pdf]
